# Informal Caregiving Relationships in Psychosis: Reviewing the Impact of Patient Violence on Caregivers

**DOI:** 10.3389/fpsyg.2018.01530

**Published:** 2018-09-03

**Authors:** Juliana Onwumere, Zheng Zhou, Elizabeth Kuipers

**Affiliations:** ^1^Department of Psychology, King's College London, Institute of Psychiatry, Psychology & Neuroscience, London, United Kingdom; ^2^Bethlem Royal Hospital, South London and Maudsley NHS Foundation Trust, Beckenham, United Kingdom

**Keywords:** psychosis, violence, aggression, carers, families

## Abstract

A modest association can be found between people with a schizophrenia spectrum diagnosis (psychosis) and perpetrating acts of violence. When a person with psychosis does engage in violence, it is their informal carers, when compared to those from the general population, who are more likely to be the targets, and violence will often occur within the family home. Despite the importance of carer support for improving patient outcomes, our understanding of how carers are impacted by patient initiated violence in psychosis remains limited. This paper reviews literature documenting the effects of patient-initiated violence in psychosis on carer functioning. The review comprised searches of Medline, PsychInfo, Embase, and Web of Science databases and the hand searches of reference lists from relevant published papers. The review was limited to English language publications from inception to 11th September 2017, and where carer experiences following reports of violence from patients with psychosis were specifically recorded. Data from 20 papers using mixed methodologies were reviewed. Patient violence in psychosis was linked to poorer carer outcomes, including carer reports of burden, trauma, fear, and helplessness. There is, however, a significant need for further studies to systematically quantify the impact and correlates of patient initiated violence on psychosis caregivers, and improve prevention.

*His mother didn't want him to be reported to the police and I was sympathetic towards that. I decided we'd do it her way, and that was a mistake, it was mistake that she paid for*.

*(Ferriter* & *Huband, 2003, p555)*

## Introduction

Schizophrenia spectrum disorders (psychosis) affect 7 per 1000 of the adult population with over 20 million people worldwide living with a diagnosis of schizophrenia alone (McGrath et al., 2008; World Health Organization, 2018). The disorders, with their first onset commonly occurring in young adulthood, are often long-term and highly burdensome (Whiteford et al., 2013). They are associated with a significantly reduced life expectancy (Hayes et al., 2017), stigma (Dickerson et al., 2002), and small social networks (Sündermann et al., 2014; Palumbo et al., 2015). Despite these challenges, many individuals with psychosis remain in close contact with informal carers from whom they receive valued support. Informal carers are a diverse group, but are mainly close family relatives (e.g., parents, partners, siblings) of patients and predominately female. The evidence base confirms that patients with carer support can achieve superior outcomes compared to peers without. The outcomes are varied but include significantly lower rates of relapse and overall number and length of psychiatric admissions (Norman et al., 2005), and improved rates of mortality (Revier et al., 2015; Ran et al., 2016) and service engagement (Stowkowy et al., 2012).

Though it has proved beneficial for improving patient outcomes, the caregiving role can impact negatively on carer health and wellbeing (Perlick et al., 2005; Flyckt et al., 2013; Gupta et al., 2015). Common mental disorders, including depression and anxiety, are significantly elevated in psychosis carers compared to the general population (Hayes et al., 2015). Carers also report experiencing exhaustion, grief reactions, and sleep disturbance (Patterson et al., 2005; Onwumere et al., 2017; Smith et al., 2018). As part of their role, many carers have also been exposed to episodes of anti-social behavior from the relatives they care for, these include episodes of both verbal and physical aggression (Belli et al., 2010; Onwumere et al., 2014).

## Psychosis and violence

Societal concerns about mental health and violence will often peak in the aftermath of a reported random act of violence (e.g., homicide) committed by an individual with mental health problems. This tends to ensure that violence, and its risk assessment, remains an important issue for mental health professionals (Shopp, 1996). Contrary to common media stereotypes, people with psychosis are more likely to have a history of victimization experiences (Bebbington et al., 2004; Honings et al., 2017), including violent victimization (Dean et al., 2007a; Short et al., 2013; ten Have et al., 2014). They also have an elevated risk for self-harm (De Hert et al., 2001). However, people with psychosis are also more likely, than the general population, to perpetrate acts of violence, including homicide (Fazel et al., 2009; Short et al., 2013). The statistical association between violence and psychosis is frequently reported as modest (Coid et al., 2006; Taylor, 2008; Douglas et al., 2009; Fazel et al., 2009), and is particularly evident during the first psychosis episode (Nielssen et al., 2007; Spidel et al., 2010; Large and Nielssen, 2011), or in some studies, during the first year of problems (Meehan et al., 2006).

Data taken from a large scale study of first episode psychosis cases in England indicated that nearly 40% of patients were aggressive at first service contact and more than half were reported as being physically violent (Dean et al., 2007b). A systematic review and meta-regression analysis of first episode psychosis highlighted that 28% of patients were aggressive prior to service contact and 31% following contact with mental health services (Winsper et al., 2013). Similarly, as part of a smaller scale study of 34 adults attending a service for people with ultra-risk psychosis mental states, 38% were reported to have had a history of violent behavior (Hutton et al., 2012). Further, approximately one fifth of adolescents meeting diagnostic criteria for psychosis and attending a community based children and young person's psychiatric service in England were recorded as having a history of physical aggression (Khalid et al., 2012).

Parallel to investigating rates of violence in psychosis, much of the research attention in this area has also focused on identifying the purported risk factors and clinical correlates of patient violence (e.g., Swanson et al., 2006; Bo et al., 2011). For example, we know that patient violence in psychosis has been positively linked to several factors. These include: younger patient age (Dean et al., 2007b; Large and Nielssen, 2011; Coid et al., 2013); substance abuse (Coid et al., 2006; Fazel et al., 2010; Spidel et al., 2010); lower educational attainment (Large and Nielssen, 2011); poor vocational activity (Swanson et al., 2006), being from an ethnic minority group (Dean et al., 2007b; Coid et al., 2013), female gender (Swanson et al., 2006); male gender (Dean et al., 2007b); social difficulties (Amore et al., 2013); history of victimization (Swanson et al., 2006; Spidel et al., 2010), and patients with a forensic history (Large and Nielssen, 2011). Violence risk has also been linked to specific symptom clusters including mania (Dean et al., 2007b; Large and Nielssen, 2011); hallucinations (Swanson et al., 2006); delusional beliefs, particularly those related to persecution, being spied upon, and conspiracy (Joyal et al., 2011; Coid et al., 2013; Onwumere et al., 2016), or where the patient perceives personal threat and/or experiences thoughts that over-ride their sense of control (Chan, 2008; Nederlof et al., 2011). Disposition to anger (Nederlof et al., 2011), particularly where anger relates to delusional beliefs, is also linked to an increased risk of violence perpetration (Coid et al., 2013; Ullrich et al., 2013). Higher rates of violence are reported in individuals with untreated psychosis (Keers et al., 2014); before commencement of pharmacological treatments (Large and Nielssen, 2008; Nielssen and Large, 2010); in patients who are non-adherent with treatments (Witt et al., 2013), and in those with a history of involuntary and/or a greater number of psychiatric inpatient admissions (Large and Nielssen, 2011; Dack et al., 2013).

When we look at other factors, we know that positive links have also been observed between patient violence and co-residence with family members (Estroff et al., 1998; Swanson et al., 2006; Kageyama et al., 2015); poorer family relationships including patient reports of not feeling listened to by their family (Swanson et al., 2006), and attempts made by carers to establish behavioral limits with patients (Straznickas et al., 1993). A review of 4,168 patients with a diagnosis of schizophrenia suggested that patients, when compared to control groups, were more likely to come to the attention of police authorities through their involvement in family violence (Short et al., 2013). Further, in approximately one third of recorded adult domestic homicides in England and Wales, over an 11 year period, perpetrators were experiencing psychotic symptoms (Oram et al., 2013).

While only a relatively small proportion of the total global population will be diagnosed with a psychotic illness, and acts of violence vary in terms of context and severity, the importance of understanding the impact of violence on a victim has remained largely ignored in the psychosis caregiving literature (Solomon et al., 2005). This is despite the fact that this is another form of domestic violence or abuse. There is a paucity of data on reports of violence from patients with psychosis who have informal carers (Thompson, 2007), and scarce consideration of the implications for its assessment and management. The limited evidence base is surprising given the contribution to and importance of the caregiving role to patient outcomes in psychosis, and the large numbers of patients who live with or continue to remain in close contact with informal carers (The Schizophrenia Commission, 2012). Lifetime rates of carer exposure to patient violence varies but has been estimated in some studies to fall within the 50–60% range (e.g., Onwumere et al., 2014; Kageyama et al., 2015), with approximately one third of carers reporting incidents of violence in the preceding year (Kageyama et al., 2015). In addition, the risk factors associated with violence perpetration within the general population (e.g., substance misuse) are also elevated in psychosis populations (Hartz et al., 2014). Further, and perhaps most compelling, is the observation that carers, particularly those who are female and living with the patient (e.g., typically mothers), are more likely to be the identified target of violent acts compared to other family members and the general population (Nordström and Kullgren, 2003a,b; Nielssen et al., 2007; Belli et al., 2010; Ural et al., 2012). Carers are also more likely to sustain greater injuries (Nordström and Kullgren, 2003a). Whilst ~8.4% of carers have issued legal orders (e.g., restraining orders) against the relatives they provide care for, following violence related issues (Solomon et al., 1995), the evidence suggests less likelihood of patient initiated violence directed toward carers ever being reported to law enforcement agencies (Nordström and Kullgren, 2003a).

## Study aims

Patient initiated violence in psychosis is an important problem for many stakeholders including family members, who are the common victims. Interpersonal violence is a public health issue that exacts a significant impact on individual wellbeing (World Health Organization, 2002). Efforts to prevent violence in family settings must commence with a more comprehensive and informed understanding of the subjective experience and impact on victims. The current paper therefore aims to review the literature on the reported effects on carers who have been exposed to violence from patients with psychosis. It specifically seeks to address the research question: *What are the reported effects of patient initiated violence on carers' physical and psychological wellbeing?* The research and clinical implications will be discussed.

## Method and terminology

### Design

A systematic review of the relevant literature with a qualitative synthesis of the findings.

### Search criteria

In accordance with the Preferred Reporting Items for Systematic Reviews and Meta-Analyses (PRISMA) statement (Moher et al., 2009), a search was undertaken of four electronic databases (Medline, PsychInfo, Embase and Web of Science) from inception to 11th September 2017. The search also included a hand search of the reference list of relevant papers to check for further applicable studies that had not been identified from the initial database review. The review was limited to: (i) studies where explicit links were reported between reports of patient initiated violence in psychosis and carer functioning; (ii) peer reviewed papers that were published in English language journals. Studies were excluded if they were review studies and those reporting data soley from non-psychosis patient populations (e.g., organic disorders like dementias), and/or psychosis conditions secondary to a primary disorder. Studies employing mixed diagnostic groups were excluded if psychotic disorders constituted < 30% of the sample.

Studies ineligible for inclusion were review studies, and those reporting data soley from non-psychosis patient populations (e.g., organic disorders like dementias) and/or psychosis conditions secondary to a primary disorder. Studies that employed mixed diagnostic groups were eligible for inclusion if psychotic disorders constituted at least 30% of the sample. Given the interchangeability in the use of terms to reflect violence (e.g., aggression) (O'Callaghan and Richman, 2010), we purposively included a broad definition of violence to account for any acts of aggression toward an individual or property, designed to threaten or inflict harm, irrespective of reported severity. A detailed list of keywords and Medical Subject Headings (MeSH) were used (with applicable search truncations and wild cards) to maximize the search capabilities and relevant paper selection. The selected terms and headings varied according to the specific database. Search terms related first to “psychosis” (serious mental illness OR severe mental disorder OR schizophren^*^ OR schizo-affective, psychosis OR psychotic), “violence” (aggression OR violence OR abuse), and carers (caregiver^*^ OR carer^*^ OR famil^*^ OR relative^*^ OR parent^*^ OR partner^*^ OR spouse^*^ OR sibling^*^). The Boolean operator “AND” was used to combine the three primary search term categories.

#### Article selection

**Figure 1:**
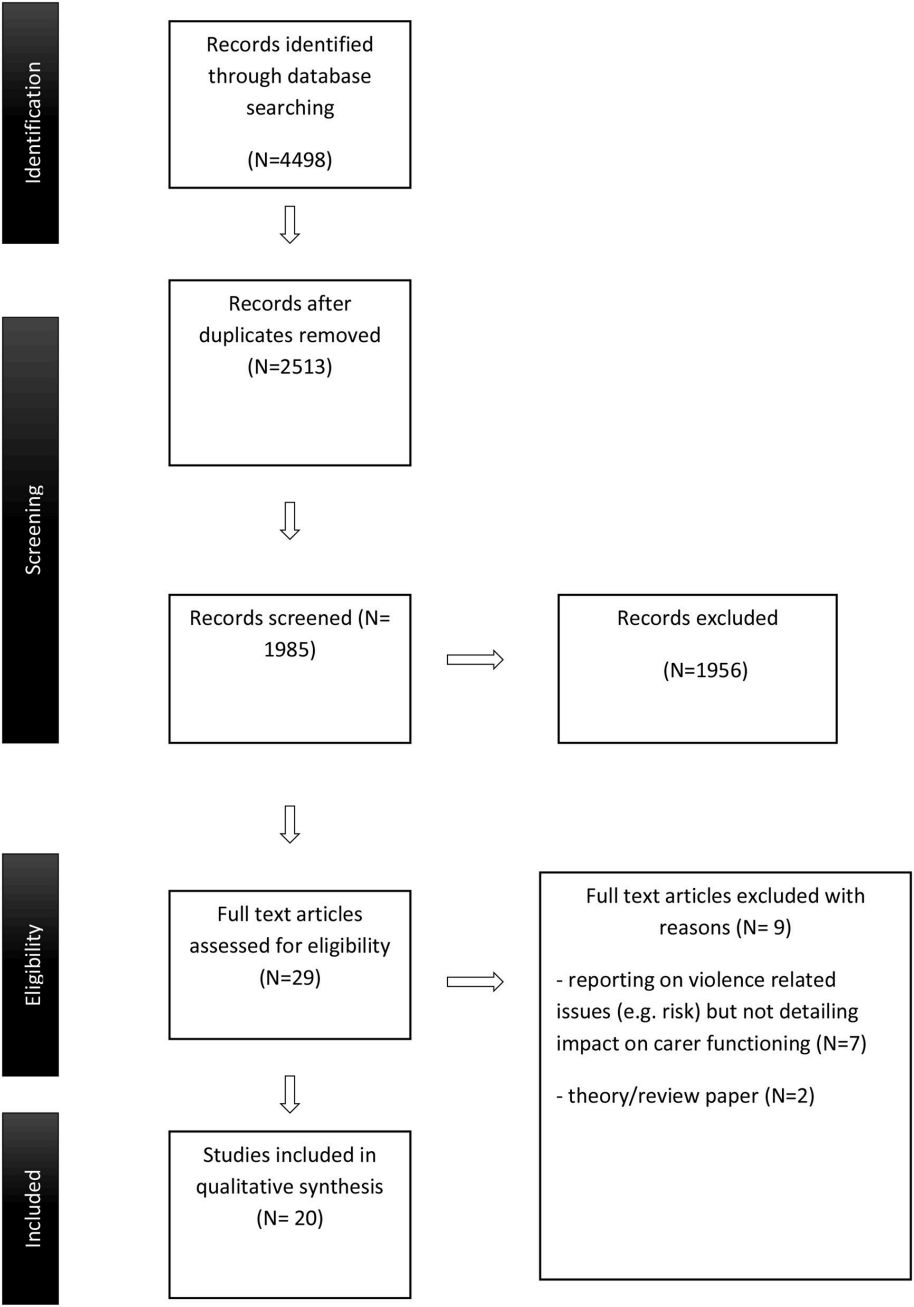
Prisma flow diagram.

The titles and abstracts of identified articles from the initial search were screened, independently, by the first two authors against eligibility criteria and to remove duplicates. Selected papers were read against inclusion criteria. Disagreement between the reviewers about a decision to include or exclude were resolved through discussion. The study selection process is outlined in Figure 1. Data from the selected studies were tabulated and presented in terms of author, publication year, study design, sample, and summary of the key findings.

## Results

The initial search strategy identified 4,498 articles, which were reduced to 2,513, following exclusion of duplicates. Twenty papers met full criteria for selection. See Table 1 for summary of studies.

**Table 1:** Summary of reviewed studies.

**Author**	**Country**	**Design**	**Violence assessment (source)**	**Time period under review for reports of violence**	**Clinical setting**	**Number of Patients (N) (% SSD)**	**Number of Carers (N) (% Female) Mean age (SD)/Range**	**Carer relationship to patient**	**Main findings**
Chan, 2008	Canada	Cross-sectional	Revised Conflict Tactics Scales (Straus et al., 1996) (Carer informant)	Preceding 12 months	Community	*N* = 51 (100%)	*N* = 61 (61% F) Mean age = 51.6 years (SD 14.1)	62.3% Parents 15% Siblings 11.5% Spouses	Positive association between patients' physical assault of carers and carer levels of burden and expressed criticism toward patients.
Chaturvedi et al., 2014	India	Cross-sectional	Scale for Assessment of Family Distress (Gopinath and Chaturvedi, 1986) (Carer informant)	NR	Community	*N* = 56 (100%)	*N* = 56 (60% F) Mean age/Range = NR	50% Parents 18% Siblings 15% Spouses	Carers reported that the most distressing patient symptoms they had to face included patients being threatening, abusive, and beating and assaulting others.
Copeland and Heilemann, 2008	USA	Cross-sectional	Open-ended interview with carer	Any occasion when patient has been violent	Community	*N* = 9 (100%)	*N* = 8 (100% F) Mean age = NR Range = 42–60 yrs	100% Mothers	Carers reported experiencing fear of their adult child, and uncertainty about what would happen next. They also reported feeling blamed for family situation troubles by authorities.
Ferriter and Huband, 2003	England	Cross-sectional	Carer semi-structured interviews Behavioral problem checklist (adapted from Kaplan and Sadock, 1989) (Carer informant)	Any time since onset of illness	Inpatient	*N* = 22 (100%)	*N* = 26 (61.5% F) Mean age = 60.8 yrs Range = 41-79 yrs	100% Parents	Patient acts of verbal aggression and violence was a common experience for carers, and was felt to contribute to levels of carer stress and burden.
Friedrich et al., 1999	USA	Cross-sectional	Impact of Illness Behaviors Scale (adapted from Lefley, 1987) (Carer informant) Open ended questions with carers	NR	NR	*N* = 22 (100%)	*N* = 15 (30% F) Mean age = 37 yrs Range = 22-52 yrs	100% Siblings	High levels of stress in carers was associated with patient episodes of abuse, which included use of weapons (e.g., guns) and throwing furniture. Carers experienced fear of future violence.
Gibbons et al., 1984	England	Cross-sectional	Social Behavior Assessment Scale (Platt et al., 1980) (Carer informant)	Preceding month	Community	*N* = 183 (100%)	*N* = 183 (% NR) Mean age = NR	40.9% Parents 46.9% Spouses 6.5% Siblings 5.46% Other	Violence, offensive behaviors, and rudeness from patients were linked to greater levels of distress in carers.
Gopinath and Chaturvedi, 1992	India	Cross-sectional	Scale for Assessment of Family Distress (Gopinath and Chaturvedi, 1986) (Carer informant)	NR	Community	*N* = 62 (100%)	*N* = 62 (39% F) Mean age = NR Range = 48% > 35 yrs	100% First degree relative or spouse	Parents did not find the aggressive or assaultive behaviors from patients as distressing, when compared to changes in other behaviors (e.g., activity related behaviors).
Hanzawa et al., 2013	Korea	Cross-sectional	Self-report questionnaire (Carer informant)	Any time since onset of illness	Inpatient & Community	*N* = 56 (100%)	*N* = 116 (55.2% F) Mean age = 55.3 yrs (SD 13.5)	54.3% Parents 25% Siblings 8.6% Children	Higher overall trauma scores (and levels of intrusion, avoidance and hyperarousal symptoms on the Impact of Events Scale—Revised, (Weiss and Marmar, 1997; Asukai et al., 2002), and burden in carers of patients with a history of violence, compared to those with no history of violence.
Hsu and Tu, 2013	Taiwan	Cross-sectional	Carer in-depth semi-structured interview	Any time since illness onset	Inpatient	*N* = 14 (100%)	*N* = 14 (57% F) Mean age = 35.7 yrs (SD NR)	100% Parents	Patient violence toward carers positively linked to carer emotional distress, fear, feelings of powerlessness in ability to control patient behavior, frustration of inability to manage the difficult situations, feelings of entrapment, and a carer's wish for child to be “normal.”
Kageyama et al., 2016	Japan	Cross-sectional	14-item checklist derived from qualitative interview with carers	Preceding 12 months	Community	*N* = 379 (100%)	*N* = 379 (67.8% F) Mean age = 69.0 yrs (SD 7.5)	100% Parents	Carers reporting high levels of psychological distress were also more likely to report experiencing greater psychological and physical violence from patients.
Kjellin and Ostman, 2005	Sweden	Cross-sectional	Carer semi-structured interview Patient medical records	Any time since the onset of illness	Inpatient	*N* = 235 (31% SSD) (43% Mood) (26% other)	*N* = 162 (51% F) Mean age = NR Range = 48% 40–59 yrs	29% Spouses 27% Parents 27% Siblings/Other relatives 12% Children	Patient violence was unrelated to carer reports of caregiving burden.
Lauber et al., 2003	Switzerland	Cross-sectional	Interview for measuring the Burden on the Family (Kluiter et al., 1998)	Any time since the onset of illness	Community	*N* = 64 (100%)	*N* = 64 (58% F) Mean age = 61 yrs (SD NR)	77% Parents	Positive association between reports of patient aggression, threats, and nuisance behavior, and carer reports of subjective and objective burden.
Loughland et al., 2009	Australia	Cross-sectional	Self–report questionnaires Perceptions of prevalence of aggression scale (POPAS, Oud, 2001).	Preceding 12 months	Community	*N* = 106 (100%)	*N* = 106 (84% F) Mean age = 54.6 yrs (SD 13.6)	49.1% Parents 32.1% Sibling 18.9% Children	23.6% of carers reported that patient aggression left them feeling that their life was in danger. 52% of carers reporting patient aggression also reported high levels of post-traumatic stress disorder (PTSD). Carers attributed blame for patient aggression firstly to the patient's mental health problems, then, to the patient, and lastly, to themselves.
Nordström et al., 2006	Sweden	Cross-sectional	Carer semi-structured interviews	None specified	Inpatient	*N* = 11 (100%)	*N* = 14 (64.2% F) Mean age = NR	64.3% Mothers 35.7% Fathers	Patient violence associated with carers experiencing fear, and distancing themselves emotionally and geographically from patient. Carers felt insecure about what their child was capable of doing.
O'Brien et al., 2006	USA	Longitudinal	Strauss Carpenter Outcomes Scale (Strauss and Carpenter, 1972)	3 months	Community	*N* = 26 ultra-high risk psychosis adolescents	*N* = 26 (84.6% F) Mean age = NR	96.1% Parents 3.8% Siblings	Patient irritability, verbal and physical aggression was associated with carers reporting criticism toward patients.
Onwumere et al., 2014	United Kingdom	Cross-sectional	Camberwell Family Interview (Vaughn and Leff, 1976)	Anytime	Inpatient and Community	*N* = 72 (100%)	*N* = 72 Mean age = 52.9 yrs (SD 12.9)	55.1% Parents 34.8% Partner 8.7% Siblings 1.4% Children	Patient violence positively linked to reports of carer hostility toward patients. Reports of patient violence positively linked to carers reporting lower self-esteem and greater use of emotion focused coping.
Thompson, 2007	USA	Cross-sectional	Carer self-report Patient self-report Patient hospital records	NR	Community	*N* = 189 (70%) Bipolar Disorder (27%)	*N* = 189 (80.9% F) Mean age = 52.2 yrs (SD NR)	54% Parents & grandparents	Patient violence and carers' experience of being victimized were associated with carers experiencing greater levels of financial strain.
Vaddadi et al., 1997	Australia	Cross-sectional	Burden on Family Interview Schedule—with adapted questions about patient violence (Pai and Kapur, 1981)	Any time since the onset of illness	Inpatient	*N* = 101 (46%)	*N* = 101 (% NR) Mean age = 47.3 yrs Range = 20-82 yrs	47% Mothers 7% Fathers 41% Partners	15% of carers reported living in fear of their relative. Positive correlation patient abusive behaviors, the number of different types of abuse, and carer distress. Carer abused by patients positively correlated with reports of carer burden including, disruptions to household routine, leisure and other relationships.
Vaddadi et al., 2002	Australia	Cross-sectional	Modified Version of Burden on Family Interview Schedule (Pai and Kapur, 1981)	Any time since the onset of illness and preceding 12 months	Community	*N* = 101 (78%)	*N* = 125 (59.2% F) Mean age = 56.9 yrs (SD 13.9)	74% Parents 13% Partner 9% Children	Carers experiencing higher levels of abuse were more likely to report emotional distress and greater burden. Higher rates of patient aggression associated with carers reporting a poorer quality relationship with patient.
Varghese et al., 2016	India	Cross- sectional	Revised Overt Aggression Scale—modified (Yudofsky et al., 1986) Aggressive Behavior and Intervention Checklist (Varghese et al., 2016)	Preceding 1 month to months	Inpatient and Community	*N* = 100 (55%) Bipolar Disorder (45%)	*N* = 100 (56% F) Mean age = 40.7 yrs (SD 13.2)	48% Parents 29% Spouse 14% Siblings 9% Children	91% reported that patient aggression negatively affected their emotions toward patient. 42% reported patient violence had led to impaired caregiving relationship. Insisting on medication adherence and discussions about the illness were common triggers to patient aggression. Carers mostly used problem focusing coping to deal with patient aggression, which involved talking calmly, and withdrawal.

*NR (Not reported)*.

### Study origin and design

The cited studies were international including six from Asia (Gopinath and Chaturvedi, 1992; Hanzawa et al., 2013; Hsu and Tu, 2013; Chaturvedi et al., 2014; Kageyama et al., 2016; Varghese et al., 2016); four from the USA (Friedrich et al., 1999; O'Brien et al., 2006; Thompson, 2007; Copeland and Heilemann, 2008); three from the United Kingdom (Gibbons et al., 1984; Ferriter and Huband, 2003; Onwumere et al., 2014) and Australia (Vaddadi et al., 1997, 2002; Loughland et al., 2009), and two from Sweden (Kjellin and Ostman, 2005; Nordström et al., 2006). The remaining studies had their origin in Canada (Chan, 2008) and Switzerland (Lauber et al., 2003). All studies, with exception of one, were cross sectional in design. The O'Brien et al. (2006) study was a 3 month longitudinal study.

### Carer demography

The total number of carer participants identified was 1,875 and most were female. In the 16 studies reporting this data, the composition of female participants in individual studies ranged from 39% (Gopinath and Chaturvedi, 1992) to 100% (Copeland and Heilemann, 2008). Only 65% (*n* = 13) of studies offered details on the mean age of carer participants. Where details were offered, carer participants were aged mainly in their early to mid-50s (Vaddadi et al., 2002; Thompson, 2007; Chan, 2008; Loughland et al., 2009; Hanzawa et al., 2013; Onwumere et al., 2014) or 60s (Ferriter and Huband, 2003; Lauber et al., 2003; Kageyama et al., 2016). There were, however, carer participants who were notably younger with their mean ages falling in the mid-30s (Friedrich et al., 1999; Hsu and Tu, 2013) and 40s (Vaddadi et al., 1997; Varghese et al., 2016).

Carer participants were heterogeneous in how they related to patients, although most were reported as being the parents. The composition of parents in individual study samples ranged from 27% (Kjellin and Ostman, 2005) to 100% (Ferriter and Huband, 2003; Nordström et al., 2006; Copeland and Heilemann, 2008; Hsu and Tu, 2013; Kageyama et al., 2016).

Carer participants also included siblings, who were sampled in 10 studies (Gibbons et al., 1984; Friedrich et al., 1999; Kjellin and Ostman, 2005; O'Brien et al., 2006; Chan, 2008; Loughland et al., 2009; Hanzawa et al., 2013; Chaturvedi et al., 2014; Onwumere et al., 2014; Varghese et al., 2016), and partners, who were reported in 9 studies (Gibbons et al., 1984; Gopinath and Chaturvedi, 1992; Vaddadi et al., 1997, 2002; Kjellin and Ostman, 2005; Chan, 2008; Chaturvedi et al., 2014; Onwumere et al., 2014; Varghese et al., 2016). Carer participants who were the children of patients were included in six studies (Vaddadi et al., 2002; Kjellin and Ostman, 2005; Loughland et al., 2009; Hanzawa et al., 2013; Onwumere et al., 2014; Varghese et al., 2016).

### Patient demography

Homogeneous schizophrenia spectrum patient samples were employed in the majority of studies (75%, *n* = 15), with a further study using a psychosis prodrome sample (O'Brien et al., 2006). Only four studies employed mixed diagnostic samples where schizophrenia spectrum diagnoses ranged from one third (e.g., 31%—Kjellin and Ostman, 2005) to ~80% of the overall sample (e.g., 78% Vaddadi et al., 2002). The additional diagnostic groups sampled were personality disorders, affective psychosis, bipolar affective disorder, and mood disorders (e.g., Vaddadi et al., 1997, 2002; Kjellin and Ostman, 2005; Thompson, 2007; Varghese et al., 2016).

### Clinical setting

Just over half of the studies sampled carers of patients who were living in community settings (Gibbons et al., 1984; Gopinath and Chaturvedi, 1992; Vaddadi et al., 2002; Lauber et al., 2003; O'Brien et al., 2006; Thompson, 2007; Chan, 2008; Copeland and Heilemann, 2008; Loughland et al., 2009; Chaturvedi et al., 2014; Kageyama et al., 2016). Inpatient only samples were used in five studies (Vaddadi et al., 1997; Ferriter and Huband, 2003; Kjellin and Ostman, 2005; Nordström et al., 2006; Hsu and Tu, 2013) and three studies used mixed inpatient and community dwelling groups (Hanzawa et al., 2013; Onwumere et al., 2014; Varghese et al., 2016). The study from Friedrich et al. (1999) did not offer any information on clinical setting.

### Assessment of violence

The operationalization of patient violence varied across studies. For example, in the Chan (2008) and Kageyama et al. (2016) studies, acts of physical, and psychological aggression were assessed. Three studies looked at acts of physical aggression only (Kjellin and Ostman, 2005; Nordström et al., 2006; Onwumere et al., 2014), with all remaining studies, but one, investigating verbal and physical aggression. It was unclear in the Copeland and Heilemann (2008) study whether verbal and/or psychological aggression was also included, in addition to physical aggression. The methods used to record data about patient violence varied considerably across studies and included patient medical records (e.g., Kjellin and Ostman, 2005; Thompson, 2007) and symptom rating scales (e.g., O'Brien et al., 2006); carer self-report questionnaires (e.g., Nordström et al., 2006; Chan, 2008; Loughland et al., 2009; Hanzawa et al., 2013; Chaturvedi et al., 2014); carer semi-structured interviews (e.g., Vaddadi et al., 2002; Lauber et al., 2003; Copeland and Heilemann, 2008; Hsu and Tu, 2013; Onwumere et al., 2014), and combinations of the different assessment methods (e.g., Gibbons et al., 1984; Gopinath and Chaturvedi, 1992; Ferriter and Huband, 2003).

For the majority of studies, the assessment period focused on any episode of patient violence that had occurred since the initial illness onset (Gopinath and Chaturvedi, 1992; Ferriter and Huband, 2003; Lauber et al., 2003; Kjellin and Ostman, 2005; Hanzawa et al., 2013; Hsu and Tu, 2013). In three studies, the assessment review period was more limited and thus focused only on reports of violence that had taken place during the preceding 12 months (Chan, 2008; Loughland et al., 2009; Kageyama et al., 2016). Two studies focused on reports during the preceding 6 months (Varghese et al., 2016) or one month (Gibbons et al., 1984), while another prospectively measured violence over 3 months (O'Brien et al., 2006). A life time prevalence of patient violence was the focus in four studies (Vaddadi et al., 1997, 2002; Copeland and Heilemann, 2008; Onwumere et al., 2014). In addition to lifetime prevalence, Vaddadi et al. (2002) also recorded reports of violence in the last 12 months. Four studies did not share any information on the assessment period under study (Friedrich et al., 1999; Nordström et al., 2006; Thompson, 2007; Chaturvedi et al., 2014).

### Impact of patient violence on carer functioning

Fourteen studies reported a positive link between patient violence and reports of carer burden (Vaddadi et al., 1997, 2002; Friedrich et al., 1999; Ferriter and Huband, 2003; Lauber et al., 2003; Chan, 2008; Hanzawa et al., 2013; Chaturvedi et al., 2014) including financial burden (Thompson, 2007), and emotional distress (Gibbons et al., 1984; Vaddadi et al., 1997, 2002; Hsu and Tu, 2013; Kageyama et al., 2016). For example, Vaddadi et al. (2002), in a sample of 101 carers, identified a positive relationship between carer reports of emotional distress on the General Health Questionnaire (Goldberg and Williams, 1988) and patient aggression. Positive links between patient violence and trauma symptoms in carers were described in two studies (Loughland et al., 2009; Hanzawa et al., 2013). Hanzawa et al. (2013) observed that carers of patients who had been violent also reported significantly higher levels of intrusion, avoidance and hyperarousal symptoms on a self-report trauma measure (i.e., Impact of Event Scale–Revised, Weiss and Marmar, 1997; Asukai et al., 2002). In contrast, the findings from Gopinath and Chaturvedi (1992) suggested that it was patient difficulties with self-care, inactivity and depressed mood that carers reported as being most distressing and not patient aggression.

In five studies, carers reported experiencing fear (Nordström et al., 2006; Copeland and Heilemann, 2008; Hsu and Tu, 2013), which included beliefs that their life was in danger (Loughland et al., 2009) and a fear of violence recurrence in the future (Friedrich et al., 1999). Data from Hsu and Tu (2013) qualitative investigation suggested that patient violence led to carer reports of feeling powerless and frustrated over their perceived inability to control patient behavior and effect positive change. Carers described making a deliberate choice to remain quiet, out of fear that their relative might retaliate (Hsu and Tu, 2013).

Three studies observed positive links between patient violence and expressed emotion, which included carer reports of patient focused criticism (O'Brien et al., 2006; Chan, 2008); hostility (Onwumere et al., 2014), and emotional over involvement (intrusiveness) (Chan, 2008). In the Vaddadi et al. (2002), patient violence was associated with carers reporting a poorer relationship between the patient and themselves.

## Discussion

Despite the fact that carers can be integral to securing optimal outcomes for those with psychosis, violence, of any type, is likely to impact negatively on any family relationship. The importance of identifying and responding to carers' individual needs has now been recognized within several clinical treatment guidelines (NICE, 2014; Galletly et al., 2016; Norman et al., 2017). The current review suggests there have been few investigations that have purposively sought to directly and systematically record the outcomes for carers who have been exposed to patient violence in psychosis. Where outcomes have been identified, patient violence is seemingly linked to a wide range of negative carer outcomes that can include burden, emotional distress, fear, and high expressed emotion (EE). These findings are offered against a body of literature which attests that poorer carer functioning and negative caregiving relationships; for example, high EE, are linked to patient poorer outcomes that include higher rates of relapse and hospitalization (Bebbington and Kuipers, 1994; Cechnicki et al., 2013; Hesse et al., 2015).

The current findings, which are based on a heterogenous group of studies, provide a useful template from which to explore, in greater detail, the carer experience of patient violence in psychosis. Though most carer participants surveyed were female, this picture is consistent with the profile of carers typically reported in psychosis research (e.g., Smallwood et al., 2017; Smith et al., 2018). In addition, the studies were diverse in country of origin and continent sampled, and their respective systems of health care provision (e.g., National Health Service; Health insurance).

It is noteworthy that the majority of studies reviewed had sampled carers at a single time point. The importance of this data should not be underestimated since it provides a much needed starting base to address pertinent questions on violence in caregiving relationships in psychosis. However, given the often repeat nature of violence, there is need to identify the potential longer-term implications of violence exposure for carer health, family outcomes, and service provision. For example, trauma presentations in carer groups are gradually receiving more research attention (Kingston et al., 2016). Exploring pathways between violence exposure, carer trauma reactions and coping styles (e.g., Loughland et al., 2009) would be beneficial and supported by multi time point studies.

Reports of patient violence toward caregivers are likely to be an underestimate, particularly when data are based upon self-report, which can be influenced by issues of social desirability (Swanson et al., 2006). Stigma and efforts to avoid adversely affecting the care and public image of their relative are likely to impact carer willingness to disclose abuse in their relationship (Kageyama et al., 2015; Onwumere et al., 2016, 2018). Further research should aim to incorporate additional sources of data; for example, accident and emergency data. Likewise, future studies exploring patient violence in caregiving relationships should seek to assess its broader impact on carer functioning and relationships, and highlight potential pathways through which patient violence may disrupt these.

## Limitations

The review was designed to offer a platform and direction for further studies but had key limitations. The selected studies tended to lack detailed information on carer participants such as weekly hours spent with patient, and the exact nature of their caregiving responsibilities; factors that could have provided more context to the findings. With exception of one, all studies were cross-sectional, thus precluding conclusions about causality. Few studies had solely set out to report on patient violence and its impact on carer outcomes, which limited the amount of data interrogation one could undertake. In accordance with the Cochrane Collaboration Tool for risk of bias (Higgins and Altman, 2008), the studies were deemed at high risk for reporting bias due to homogeneity in methods used to record outcomes on carer impact (i.e., self-report). Finally, it is unclear to what extent the current findings are specific to psychosis caregivers or are observed in other severe mental health caregiver groups such as bipolar affective disorder. Future reviews may wish to extend the population group under study to include other diagnostic groups, which can help in the process of determining the scale of the problem and assessing the need for psychosis specific or trans-diagnostic responses.

## Implications

The cognitive model of caregiving responses in psychosis highlights the importance of carer appraisals about the patient and the illness on overall outcomes (Kuipers et al., 2010). Though much has been written about issues of domestic violence and mental health (Howard, 2012), there has been a noticeable neglect of these issues when they are reported by informal carers of people with psychosis. For policy makers, greater awareness of the different family settings that interpersonal violence can and does occur, and the additional unique and complex needs faced by informal carers is required. A consideration of nuanced and targeted informant campaigns, specifically designed for those in caregiving roles and with an understanding of the broader issues should be given. More research is required to improve our understanding of the impact of patient violence on carer outcomes and the implications for their caregiving relationship. The data should help to facilitate the development of tailored interventions for carers and patients to help prevent such problems, minimize the risk of patient violence and the potential negative psychological and physical sequelae for carers. Recent findings from Bowman et al. (2014) suggest that the quality of life in the siblings of early psychosis patient groups can be negatively affected by a patient's history of violence. Thus, exploring the needs of other family members who may not be in primary caregiver roles but are nevertheless affected by patient violence would seem a helpful way forward. In services amongst clinicians, more efforts are required to routinely and systematically record patient violence in caregiving relationships and its impact. Developing carer focused support interventions, which could be incorporated into community treatment models, are likely to benefit both carer and patient outcomes (McCann et al., 2011), and are consistent with recommended treatment approaches (NICE, 2014, 2015). Though most adults with psychotic disorders do not engage in violence, domestic violence in psychosis should be an issue of public health and concern. Focusing on building a better understanding of the patient sub groups who engage in acts of violence toward their caregivers might support the development of preventative and targeted interventions, which would have the potential to improve outcomes for all.

## Conclusion

In psychosis, our findings indicate that patient violence in caregiving relationships can impact carer wellbeing and outcomes. Historically, however, carer needs and their issues have tended to be overlooked and marginalized. The current findings underscore the importance of focusing clinical and research efforts on carers and caregiving relationships affected by patient violence.

## Author contributions

JO led on the conceptualization and design of the project. JO and ZZ led on the database searches and data synthesis. All authors contributed to preparing the manuscript.

### Conflict of interest statement

The authors declare that the research was conducted in the absence of any commercial or financial relationships that could be construed as a potential conflict of interest.
